# Changes in γH2AX and H4K16ac levels are involved in the biochemical response to a competitive soccer match in adolescent players

**DOI:** 10.1038/s41598-020-71436-6

**Published:** 2020-09-02

**Authors:** Katarzyna Kozioł, Jacek Zebrowski, Gabriela Betlej, Ewelina Bator, Wojciech Czarny, Wojciech Bajorek, Bartłomiej Czarnota, Robert Czaja, Paweł Król, Aleksandra Kwiatkowska

**Affiliations:** 1 Department of Animal Physiology and Reproduction, University of Rzeszów, Werynia 502, 36-100 Kolbuszowa, Poland; 2 Institute of Biology and Biotechnology, University of Rzeszów, Aleja Rejtana 16c, 35-959 Rzeszów, Poland; 3 Laboratory of Exercise Physiology and Biochemistry, Institute of Physical Culture Studies, College of Medical Sciences, University of Rzeszów, Aleja Rejtana 16c, 35-959 Rzeszów, Poland; 4 Institute of Physical Culture Studies, College of Medical Sciences, University of Rzeszów, Aleja Rejtana 16c, 35-959 Rzeszów, Poland; 5 Department of Swimming Sports, Institute of Physical Culture Studies, College of Medical Sciences, University of Rzeszów, Aleja Rejtana 16c, 35-959 Rzeszów, Poland

**Keywords:** Biophysical chemistry, Cytokines, DNA

## Abstract

The aim of this study was to examine novel putative markers of the response to the competitive soccer match in adolescent players, such as changes in global levels of γH2AX and H4K16ac in the chromatin of peripheral mononuclear blood cells (PMBCs) and a Fourier-transform infrared spectroscopy (FTIR)-based biochemical fingerprint of serum. These characteristics were examined with reference to the physiological and metabolic aspects of this response. Immediately post-match we noticed: (1) a systemic inflammatory response, manifesting as peaks in leukocyte count and changes in concentrations of IL-6, TNFα, and cortisol; (2) a peak in plasma lactate; (3) onset of oxidative stress, manifesting as a decline in GSH/GSSG; (4) onset of muscle injury, reflected in an increase in CK activity. Twenty-four hours post-match the decrease in GSH/GSSG was accompanied by accumulation of MDA and 8-OHdG, macromolecule oxidation end-products, and an increase in CK activity. No changes in SOD1 or GPX1 levels were found. Repeated measures correlation revealed several associations between the investigated biomarkers. The FTIR analysis revealed that the match had the greatest impact on serum lipid profile immediately post-game. In turn, increases in γH2AX and H4K16ac levels at 24 h post-match indicated activation of a DNA repair pathway.

## Introduction

Soccer is an intermittent sport characterized by very demanding, high-intensity actions (i.e. sprints, dribbling, jumping, shots) that occurs in bursts over a period of low-intensity activity (i.e. jogging, walking or even brief recovery intervals)^1^. As such it cannot be classified as a pure aerobic or pure anaerobic activity and would not be considered resistance training regardless. It represents a mixture of these elements, which together, especially under competitive match conditions, induces a multi-level, acute response in many physiological systems including the hematological, hormonal, musculoskeletal, metabolic and immune systems that is reflected in changes in metabolic and biochemical profiles^1–3^. Therefore, soccer game-induced physiological and biochemical changes can be considered a model of whole-body somatic stress^4^.

First of all, strenuous physical activity has an impact on the hematological system, which manifests as changes in several parameters, mainly leukocyte count^5^ and, in many cases, plasma volume expansion^6–8^. In addition to transient leukocytosis^5^, the systemic inflammatory response^9^ is also reflected in increases in early mediators of exercise-induced inflammation, such as circulating interleukin IL-6 and TNFα^10–12^. In addition, a rise in the concentration of cortisol, an immunomodulatory hormone, is thought to contribute to the inflammatory response^13^.

Second, high-intensity activity that depends mainly on anaerobic metabolism results in muscle fatigue, which manifests as, for example, an increase in serum lactate concentration and ATP depletion^14^, which means that on-going physical activity is dependent on aerobic regeneration of ATP^15^. However, enhanced oxygen uptake can induce oxidative stress, defined as an imbalance between reactive oxygen species (ROS) and the effectiveness of the antioxidant defense systems, with the former becoming more dominant. The oxidative stress-driven disturbances in the pro- and anti-oxidant homeostasis of cells are reflected in changes in the redox status of glutathione, a widely used indicator of both oxidative stress^16^ and physical overtraining^17^. Glutathione is part of the non-enzymatic complex^16^, whereas antioxidant enzymes, such as superoxide dismutase (SOD) and glutathione peroxidase (GPX) are components of the antioxidant enzymatic system^18^.

The toxic effects of raised levels of ROS include oxidative modifications of macromolecules such as lipids, proteins and DNA. One of the consequences of oxidative attack is lipid peroxidation, a multistep process resulting in structural and functional changes to cell membranes and lipid-containing molecules. Arachidonic acid-derived malondialdehyde (MDA) is one of the end products of oxidative stress-associated lipid peroxidation and serves as a marker of oxidative damage to lipids. In turn, lipid peroxidation-dependent modifications of cell membranes can change their permeability, resulting in an efflux of intracellular creatine kinase (CK) from muscle^19^. Hence extracellular serum/plasma CK activity is another commonly used marker of the response to muscle damage following strenuous physical activity^20^.

The wide range of oxidative stress-driven DNA modifications includes the formation of 8-hydroxy-2′-deoxyguanosine (8-OHdG), the most common product of oxidative damage to guanine^21^, and double-strand DNA breaks (DSBs). The latter are considered the most hazardous form of DNA damage. In physiological conditions, however, DSBs are repaired, a process that is preceded by signaling the presence of DNA damage and involves enrichment of phosphorylation on serine 139 of the histone variant H2A.X (this phoshorylated isoform of H2AX histone is denoted as γH2AX) at the nucleosomes surrounding DSBs, which is accompanied by an increase in acetylation on serine 16 of histone H4 (H4K16ac)^22,23^. To date few studies have focused on the epigenetic mechanisms associated with biochemical pathways induced by intense physical activity^24^ and even less is known about the potential role of chromatin modifications in fine-tuning these responses. In particular, there is still lack of information concerning epigenetic aspects of the exercise-induced DNA damage repair (DDR) pathway, especially in the sport science field.

Therefore, the aim of this study was to examine novel biomarkers, including epigenetic markers such as alterations in global levels of phoshorylation of the H2AX histone and acetylation of H4K16 in the chromatin of peripheral mononuclear blood cells (PMBCs) as well as to carry out attenuated total reflectance Fourier transform infrared spectroscopy (ATR-FTIR)-based analysis of serum in adolescent soccer players following a high-level competitive soccer match. These epigenetic markers and biochemical characteristics were examined in relation to a wide range of biomarkers contributing to physiological response to a soccer game, including markers related to hematological changes, inflammation, oxidative stress, muscle fatigue and muscle cell injury.

## Results

### Alterations in hematological parameters of the soccer players in response to a match

Table 1 summarizes changes in the subjects’ hematological parameters throughout the observation period. The most dramatic change in the variables investigated occurred in leukocyte count, which increased 2.45 (± 0.46) times immediately after the match and declined almost to baseline values over the next 24 h (Table 1).

**Table 1:** Soccer match-induced changes (mean ± SD) in hematological parameters of the players (N = 10).

Parameter	Pre-match	Immediately post-match	24 h post-match
White blood cell (WBC) [10^3^/μL]	5.89 (± 1.36)	14.26 (± 3.83) a	6.14 (± 1.82) b
Red Blood Cell (RBC) [10^6^/μL]	5.29 (± 0.28)	5.11 (± 0.21) a	5.03 (± 0.19) a
Hemoglobin (Hb) [g/dL]	15.75 (± 0.66)	15.21 (± 0.55) a	14.95 (± 0.52) a
Hematocrit (Hct) [%]	46.79 (± 2.07)	44.82 (± 1.67) a	44.84 (± 1.69) a
Platelet (PLT) [10^3^/μL]	240 (± 33.65)	249.9 (± 41.62) a	228.3 (± 37.32) a
Mean corpuscular volume (MCV) [fL]	88.44 (± 1.89)	87.67 (± 1.6) a, b	89.17 (± 1.67) a, b
Mean corpuscular hemoglobin (MCH) [pg]	29.77 (± 0.72)	29.77 (± 0.78)	35.62 (± 18.45)
Mean corpuscular hemoglobin concentration (MCHC) [g/dL]	33.68 (± 0.69)	33.94 (± 0.52)	33.34 (± 0.45)
Plasma volume changes [%]		+ 7.49 a	+ 9.39 a

a indicates a difference (*p* < 0.05) between mean values immediately post-match relative to pre-match mean values; b indicates a difference (*p* < 0.05) between mean values post-match and 24 h post-match.

Immediately post-match and after 24 h recovery we also observed decreases in hemoglobin (Hb) and hematocrit (Hct) values, which were accompanied by slight, non-significant alterations in total red blood volume. Together these changes resulted in increases in plasma volume, relative to mean pre-match values, of about 7.5% and 9.4% immediately post-match and 24 h post-match, respectively (Table 1).

We also observed a decrease in red blood cell count at both post-match measurement points, whilst mean corpuscular volume (MCV) value was lower immediately post-match and elevated after 24 h of recovery. Platelet count showed the opposite pattern, being elevated immediately post-match and reaching its lowest value after 24 h recovery.

### Changes in levels of inflammation-related markers, cortisol and lactate

**Figure 1:**
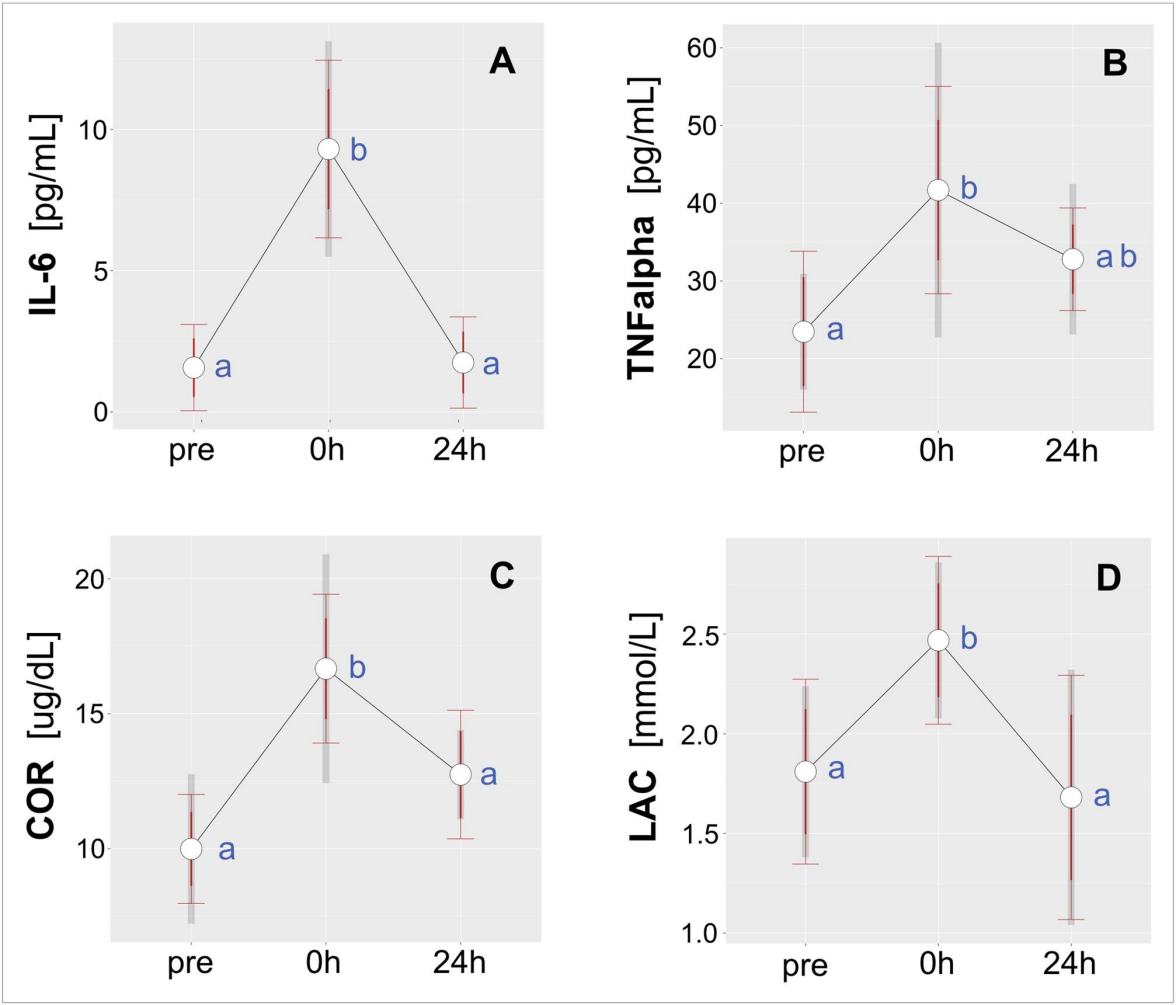
Effect of a competitive soccer match on the biochemical characteristics of serum collected before (pre), immediately after (0 h) and 24 h later (24 h): (**A**) IL-6, interleukin-6, [pg/mL]; (**B**) TNFα, tumor necrosis factor α, [pg/mL]; (**C**) COR, cortisol, [µg/dL]; and (**D**) LAC, lactate, [mmol/L]. The red error bars indicate 95% confidence intervals calculated at within-subject level and adjusted according to Cousineau^67^ and Morey^68^. The grey bars represent 95% confidence intervals for between-subjects variability. Different letters indicate significant differences between means (*p* < 0.05).

Peaks in serum concentrations of IL-6, TNFα and cortisol were observed immediately after the match. We observed 5.94 (± 3.4)-fold, 1.25 (± 0.47)-fold, and 1.78 (± 0.65)-fold post-match increases in IL-6, TNFα and cortisol respectively, and values returned to baseline by 24 h post-match (Fig. 1A–C, respectively).

Similarly, plasma lactate concentration peaked immediately post match [1.45(± 0.65)-fold increase relative to pre-match level] and returned to its baseline value at 24 h post-match (Fig. 1D).

### Changes in CK activity and alterations in oxidative stress-related markers

**Figure 2:**
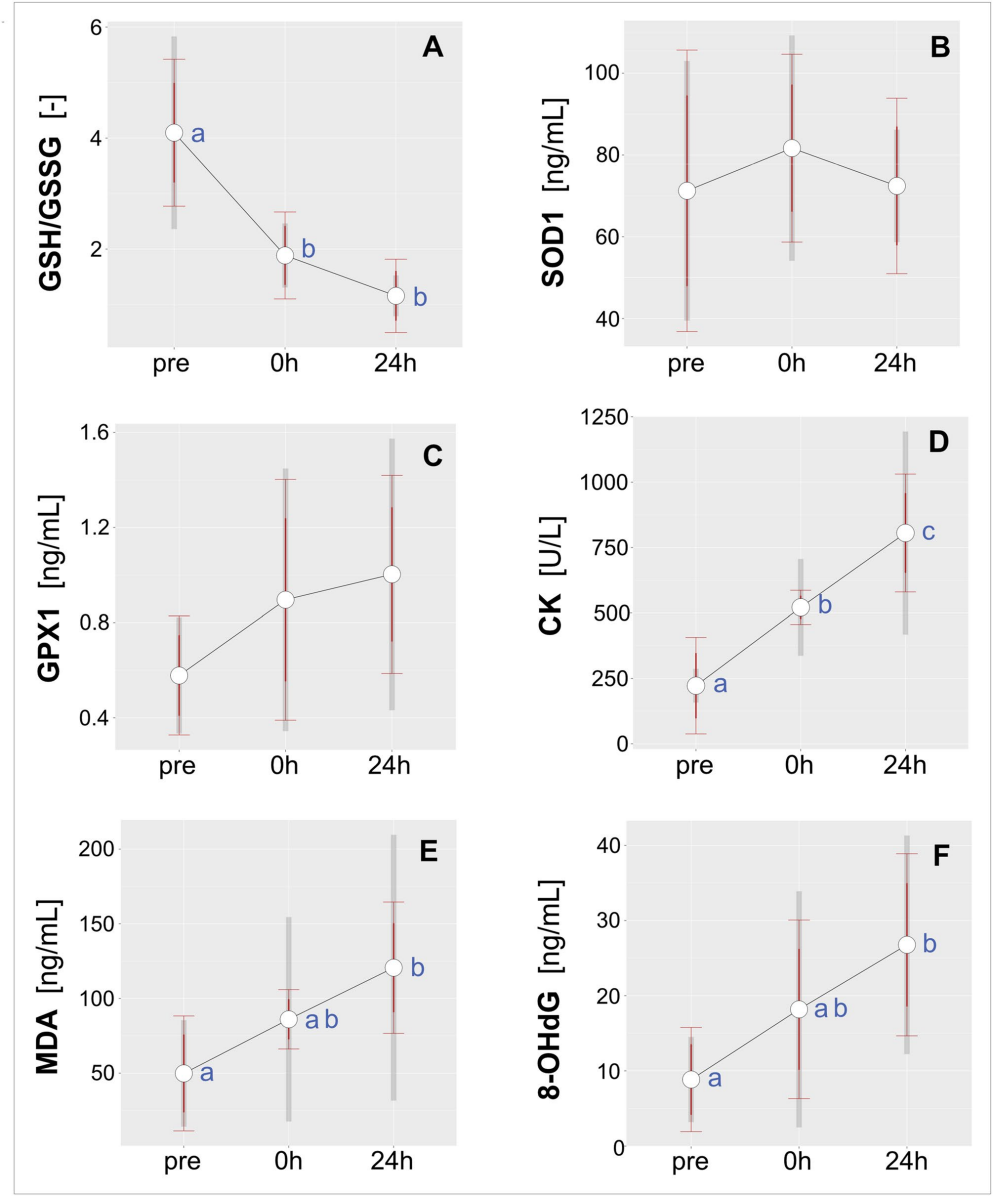
Effect of a competitive soccer match on the biochemical characteristics of serum collected before (pre), immediately after (0 h) and 24 h later (24 h): (**A**) GSH/GSSG, GSH (reduced glutathione)/GSSG (oxidized glutathione) ratio; (**B**) SOD1, Cu/Zn superoxide dismutase, [ng/mL]; (**C**) GPX1 [ng/mL]; (**D**) CK, creatine kinase activity, [U/L]; (**E**) MDA, glutathione peroxidase 1, [ng/mL]; and (**F**) 8-OHdG, 8-hydroxy-2′-deoxyguanosine, [ng/mL]. The red error bars indicate 95% confidence intervals calculated at within-subject level and adjusted according to Cousineau^67^ and Morey^68^. The grey bars represent 95% confidence intervals for between-subjects variability. Different letters indicate significant differences between means (*p* < 0.05).

Immediately after the match we observed decreases in the level of reduced glutathione (GSH) and an accumulation of its oxidized form (GSSG), reflected in a decline in GSH/GSSG ratio that persisted over the next 24 h (Fig. 2A). We observed 2.33 (± 1.32)-fold and 4.42 (± 3.45)-fold decreases in GSH/GSSG ratio relative to baseline. There were no changes in serum levels of SOD1 (Fig. 2B) and GPX1 (Fig. 2C). However, we observed a progressive increase in serum CK activity following the soccer match: it was 2.33(± 0.48) times higher than baseline immediately post-match and 3.43 (± 1.2) times higher after 24 h of recovery (Fig. 2D). Twenty-four hours post-match there was 1.45(± 0.4)-fold increase relative to mean values reported immediately after the match (Fig. 2D). Additionally, the soccer match had an impact on MDA and 8-OHdG 24 h post-match, with increases of 2.83 (± 1.46) times and 5.69 (± 7.18) times respectively, relative to pre-match values (Fig. 2E,F, respectively).

### Associations between the investigated biochemical markers

**Figure 3:**
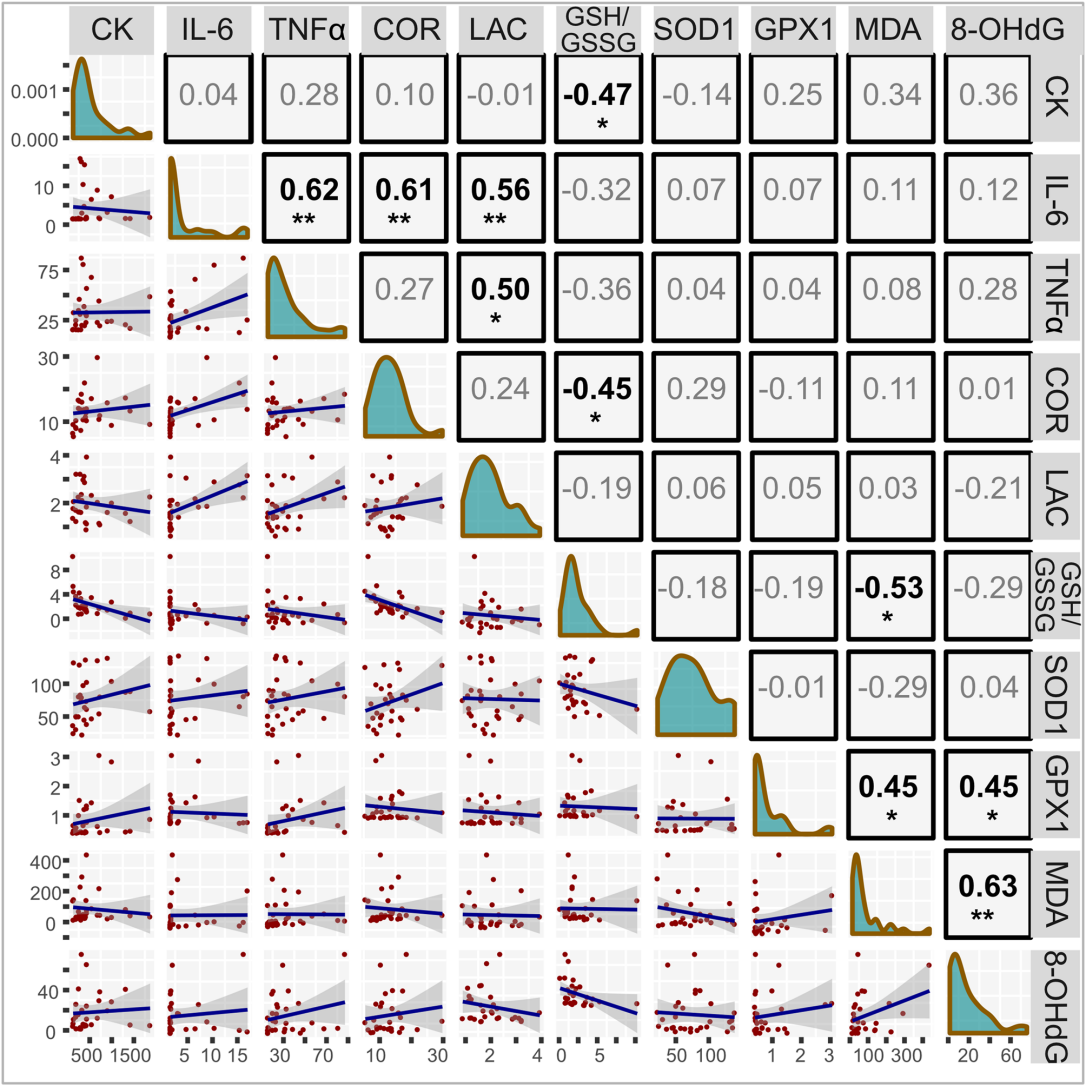
Descriptive analysis of serum properties. The lower triangle shows scatterplots of overall data with linear regression lines and confidence intervals for each combination of variables. The diagonal panels present density distributions of the examined features. The upper diagonal constitutes the correlation matrix with repeated measures correlation coefficients. The p-values were calculated on the basis of bootstrapping procedures. (CK) creatine kinase activity [U/L]; (IL-6) interleukin 6 [pg/mL]; (TNFα) tumor necrosis factor α [pg/mL]; (COR) cortisol [µg/dL]; (LAC) lactate [mmol/L]; (GSH/GSSG) GSH (reduced glutathione)/GSSG (oxidized glutathione) ratio; (SOD1) Cu/Zn superoxide dismutase [ng/mL]; (GPX1) glutathione peroxidase 1 [ng/mL]; (MDA) malondialdehyde [ng/mL]; (8-OHdG) 8-hydroxy-2′-deoxyguanosine [ng/mL].

Repeated measures correlation analysis (rmcorr) revealed several associations between the investigated biomarkers. There were large positive correlations between: IL-6 and TNFα (r = 0.62, *p* = 0.003), IL-6 and cortisol (r = 0.61, *p* = 0.003), IL-6 and lactate (r = 0.56, *p* = 0.08), TNFα and lactate (r = 0.5, *p* = 0.02), MDA and OHdG (r = 0.63, *p* = 0.002), a moderate positive correlation between GPX1 and 8-OHdG (r = 0.45, *p* = 0.042), a large negative correlation between MDA and GSH/GSSG (r = − 0.53, *p* = 0.014) and moderate negative correlations between CK and GSH/GSSG (r = − 0.47, *p* = 0.031) and cortisol and GSH/GSSG (r =  − 0.45, *p* = 0.04). All results are summarized in Fig. 3.

**Figure 4:**
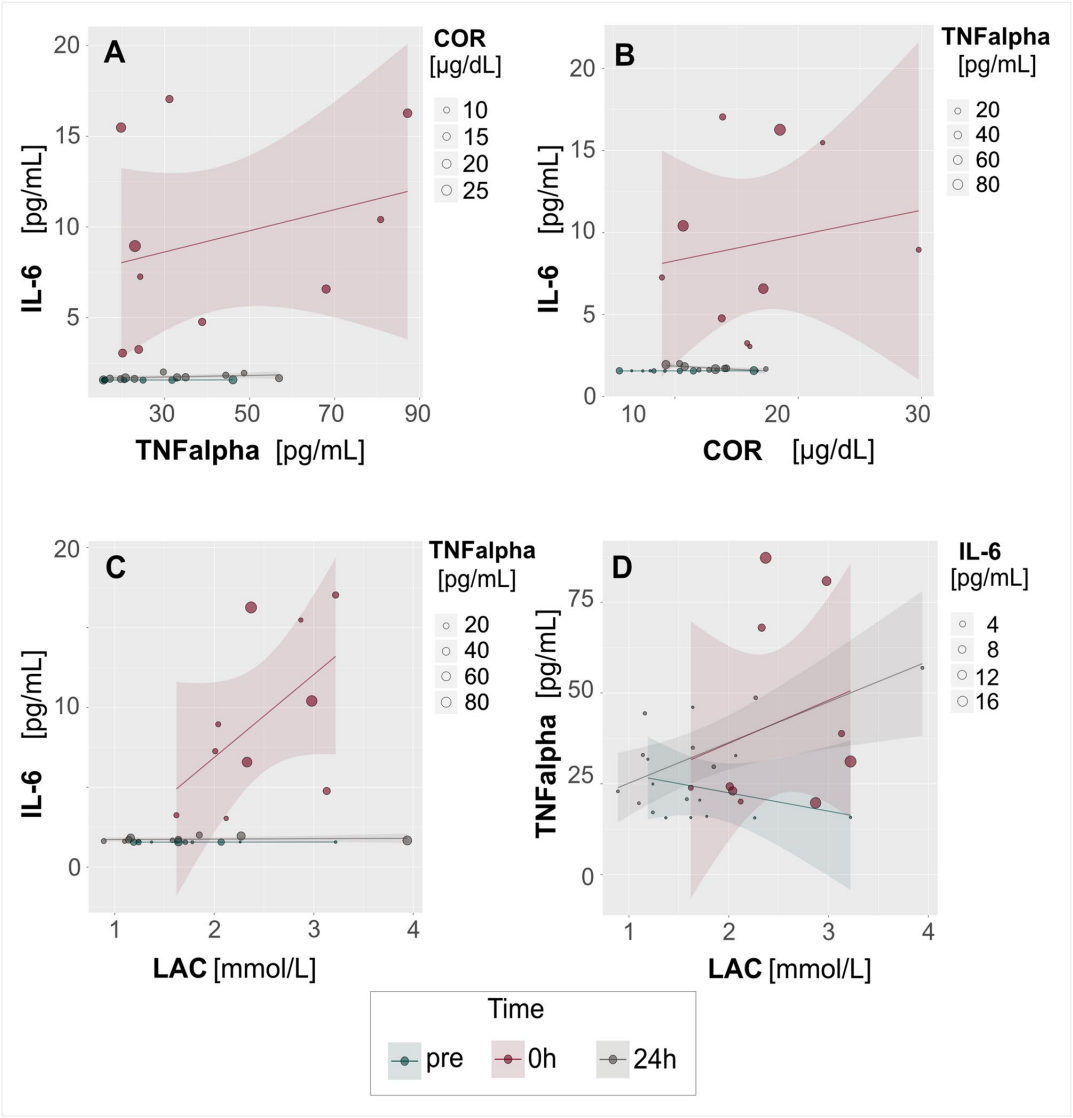
A bubble plot with multiple groups (three time-points) displaying values of three variables at a time. Two variables are plotted on the axes as in a typical scatterplot, whilst the third variable is depicted using circles of varying size. Linear regression lines with confidence intervals have been added for each group of data to show general trends in variability and interdependence. Panels **A**–**C** shows that response to effort as indicated by IL-6 was higher at high levels of (**A**) TNFα; (**B**) COR; and (**C**) LAC. Neither pre-match nor 24 h post-match was there any relationship between these variables. (**D**) TNFα was positively correlated with LAC immediately after the match and this relationship was unchanged after 24 h; before the match these variables were negatively related. (CK) creatine kinase activity, [U/L]; (IL-6) interleukin-6, [pg/mL]; (TNFα) tumor necrosis factor α, [pg/mL]; (COR) cortisol, [µg/dL]; (LAC) lactate, [mmol/L].

Looking in more detail we note that the large positive correlations between IL-6 and TNFα (Fig. 4A), IL-6 and cortisol (Fig. 4B) and IL-6 and lactate (Fig. 4C) were only observed immediately post-match. There pairs of variables were not correlated before the match or 24 h after it (Fig. 4A–C). In the case of TNFα and lactate, there was a large positive correlation both immediately post-match and at 24 h post-match (Fig. 4D).

**Figure 5:**
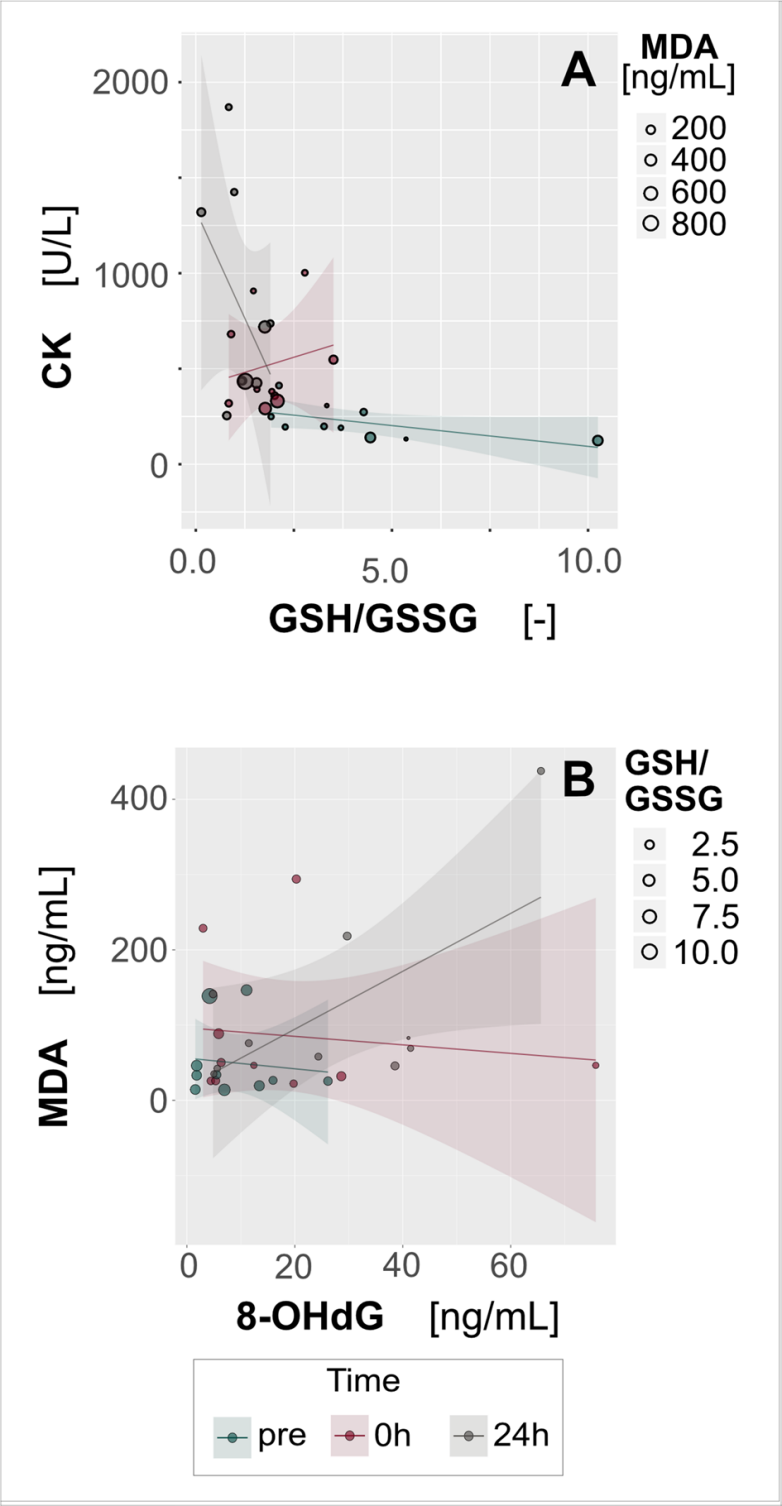
A bubble plot with multiple groups (three time-points) displaying values of three variables at a time. Two variables are plotted on the axes as in a typical scatterplot, whilst the third variable is depicted using circles of varying size. Linear regression lines with confidence intervals have been added for each group of data to show general trends in variability and interdependence. There was no clear relationship between (**A**) CK and GSH/GSSG before or immediately after the match, but 24 h after the match CK level was negatively correlated with GSH/GSSG ratio. (**B**) MDA was not related to 8-OHdG before or immediately after the match, but 24 h after the match these two variables were positively correlated. (CK) creatine kinase activity, [U/L]; (GSH/GSSG) GSH (reduced glutathione)/GSSG (oxidized glutathione) ratio; (MDA) glutathione peroxidase 1, [ng/mL]; (8-OHdG) 8-hydroxy-2′-deoxyguanosine, [ng/mL].

At 24 h post-match we observed a moderate negative correlation between CK and the GSH/GSSG ratio (Fig. 5A) as well as a large positive correlation between MDA and 8-OHdG values (Fig. 5B). These variables were not correlated at the pre-match or immediate post-match time-points.

### FTIR-based analysis of serum following the soccer match

**Figure 6:**
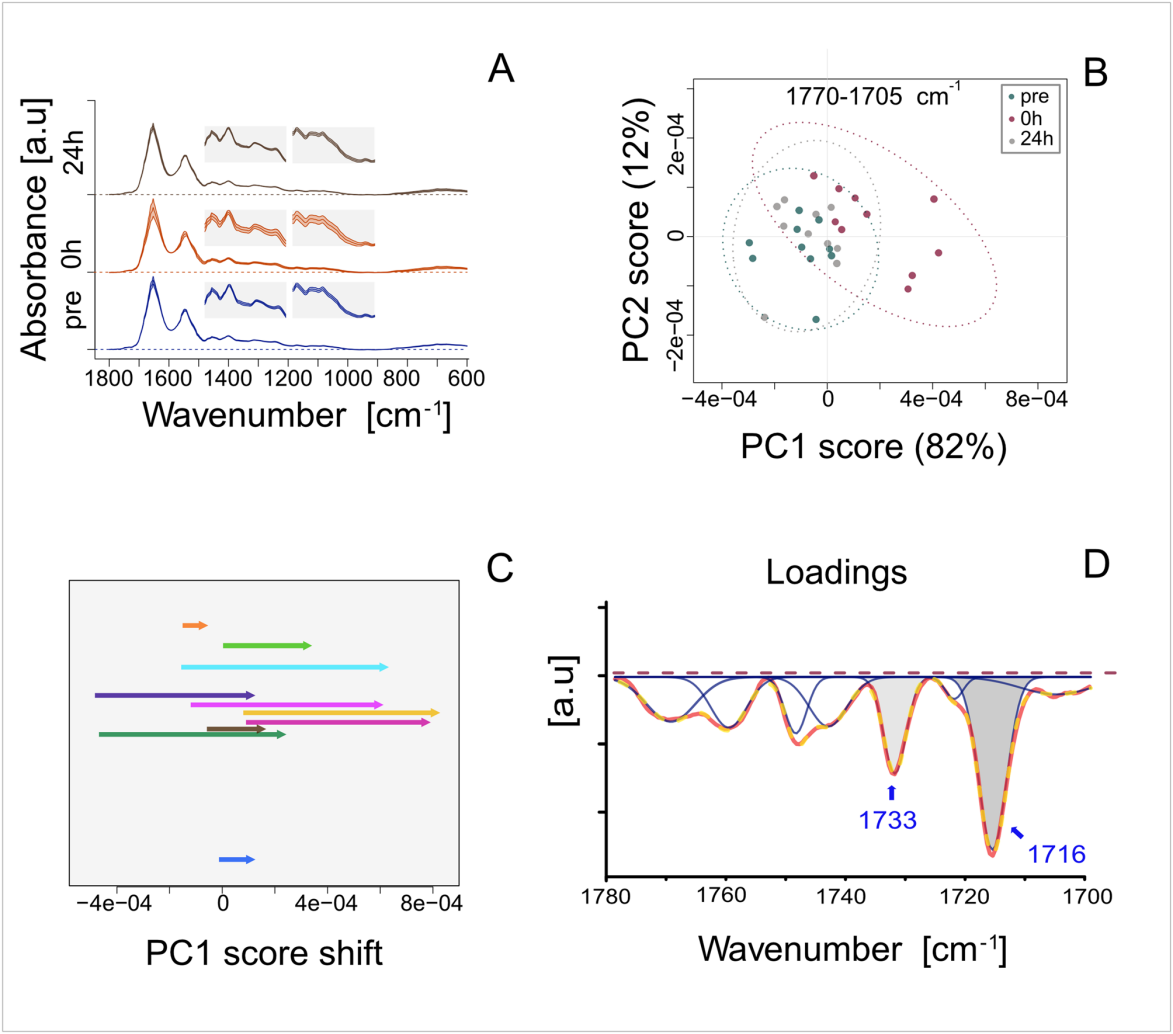
FTIR analysis of blood serum. (**A**) Mean ± SD of spectra collected between 1,800 and 600 cm^−1^ wavenumbers for before a soccer match, immediately after and 24 h after. The 1,500–1200 cm^−1^ and 1,200–900 cm^−1^ regions have been enlarged for clarity and are presented as the insets. (**B**) Principle component analysis (PCA) performed on the spectral region between 1,770 and 1705 cm^−1^. The PC2 scores are plotted vs PC1 scores; they explain 12% and 82% of total variability, respectively. Confidence intervals (95%) are marked as ellipses. (**C**) The match-related shift in PC1 scores for particular individuals (pre-match were used as the reference). (**D**) The loading factor plot for PC1 indicates the contribution of various spectral bands to the clustering pattern. The loading curve was local baseline corrected, and Gaussian deconvolution of overlapped peaks was performed to extract distinct compounds (blue). The sum of the fitted curves (dashed line) closely covered the experimental data (solid red line).

Infrared spectra of serum from the blood collected pre-match, immediately post-match and 24 h post-match are shown in Fig. 6A as means ± SD. They show the prominent amide I (around 1650 cm^−1^) and amide II (around 1550 cm^−1^) bands typical of serum and a complex fingerprint region (1,500–900 cm^−1^) with multiple overlapping peaks. The general pattern of the spectra did not change as the result of the effort; however the inter-subject dispersion of the spectra absorbance was higher immediately after the match (relative to the pre-match value) but decreased again over the following 24 h. This is reflected in the SD values, presented graphically as the insets in Fig. 6A. The enhanced inter-subject variability shortly after the match may indicate subtle but complex, reversible changes in the biochemical profile of the serum. Moreover, the data indicate that there is high inter-subject variability in this aspect of the response to exercise.

The spectral data for selected regions (specified in the methods) were subjected to multivariate analysis (PCA) to reduce the data dimension and to establish possible clustering patterns of the samples. The most interesting results, showing clear distinction of samples, were found for the spectral region between 1,800–1,700 cm^−1^ (Fig. 6B). For the PC1 scores, which explained 82% of total variability of the data, samples collected immediately after the effort displayed more positive values compared to remained groups. Figure 6C indicates the shift toward higher PC1 values was observed for every participant. In order to reveal which wave numbers—and thus which corresponding chemical compounds—contributed to the observed variability of the data and the sample discrimination, we performed loading factor analysis. The loading factor plot showed overlap of multiple bands (data not shown). To resolve this overlap into compound peaks, we performed local baseline correction and deconvolution processing using Gaussian shape (Fig. 6D). The bands corresponding to compounds with C=O in a free carboxylic acid and or in an ester functional group were centered at 1716 and 1733 cm^−1^, respectively.

### Changes in global levels in γH2AX and H4K16 acetylation

**Figure 7:**
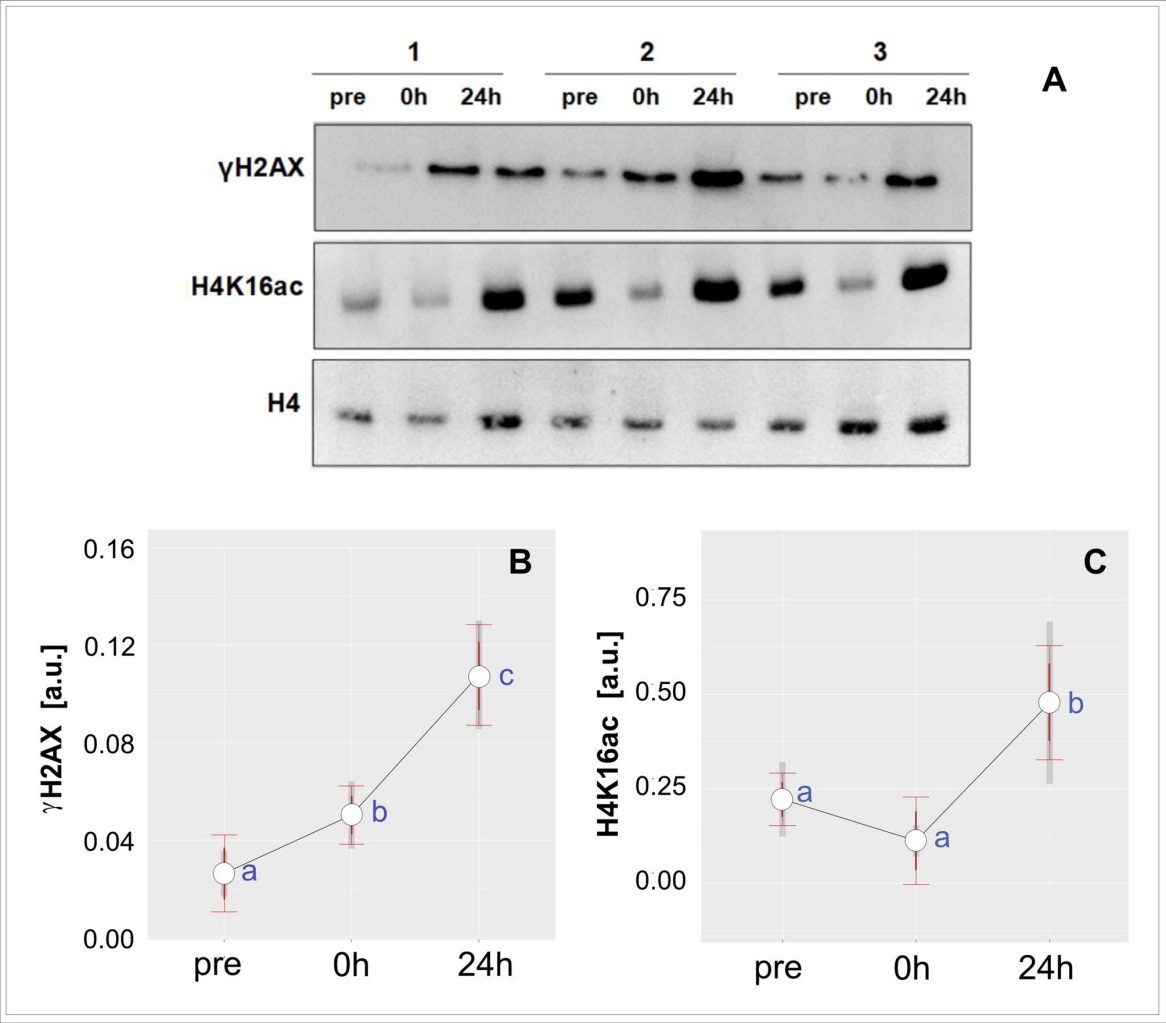
Effect of a competitive soccer match on the changes in global levels of γH2AX and H4K16ac in the PMBC chromatin collected before (pre), immediately after (0 h) and 24 h later (24 h) for three representative participants (designed as 1, 2, and 3). (**A**) The cropped blots are presented and their full-length blots are included in the Supplementary information Fig. S1. The corresponding full-length SDS-PAGE gel is included in the Supplementary information Fig. S2. (**B**,** C**) The relative levels of (**B**) γH2AX and (**C**) H4K16ac. Western blots were quantified by densitometry (using ImageJ program), normalized to H4 histone (a loading control as described elsewhere^59^). The relative γH2AX (**B**) and H4K16ac (**C**) protein levels are shown. All samples derived from the same experiment and blots were processed in parallel. The red error bars indicate 95% confidence intervals calculated at within-subject level and adjusted according to Cousineau^67^ and Morey^68^. The grey bars represent 95% confidence intervals for between-subjects variability. Different letters indicate significant differences between means (*p* < 0.05).

Immediately after the match we observed a decrease in the global level of H4K16ac, followed by its significant increase at 24 h post-match relative to baseline (Fig. 7). In the case of γH2AX, we reported a progressive increase in its level throughout 24 h of recovery (Fig. 7).

## Discussion

The main aim of this study was to examine the influence of a competitive soccer match on the acetylation status of histone H4 and the γH2AX protein level in PBMC from adolescent players. To the best of our knowledge, this is the first study concerning those aspects of the biochemical response to a soccer game. We focused on those epigenetic markers for two reasons. First, although the relationship between exercise and DNA damage has been addressed in several studies^24,25^, there is still lack of information concerning epigenetic aspects of the exercise-induced DDR pathway, especially in the sport science field. Second, the data for this study were collected under real competitive conditions, in the presence of a wide range of environmental stressors. In such a context, the epigenome-mediated adaptive response has a special role in fine-tuning an organism's metabolism and homeostasis^27^. Given the fact that those epigenetic components are only a single aspect of the whole-body response to a soccer match, we examined a wide range of different biomarkers that reflected the involvement of the corresponding organism's systems to such a response. Furthermore, we also intended to provide a broader physiological and biochemical context for presenting novel aspects of the analyzed response. To adopt a more holistic approach, we investigated the impact of a soccer match on the measured variables, as well as their mutual interrelations, by performing a wide array of correlations between them. Finally, to complete the overall view of soccer match–induced biochemical changes, we performed FTIR-based fingerprinting of the whole serum to establish interesting spectral markers associated with the soccer effort; these data are valuable for the present and future studies.

Overall, immediately after a soccer match, we observed a rapid but transient systemic inflammatory response as well as an increase in plasma lactate concentration, Besides their canonical roles, these factors may also play a more general role in metabolism, given the pleiotropic function of IL-6 and TNFα^11–13^ as well as lactate^28^. These metabolic changes were accompanied by alterations in the lipids biochemical profile that—as FTIR-based fingerprint of serum revealed—involves changes in the levels of triglycerydes (TG) and free fatty acids (FFA). In turn, 24 h post-match, the long-term consequences of oxidative stress and deployment of mechanisms for counteracting oxidative stress–related effects commenced, including activation of the DDR pathway, as denoted by epigenetic markers such as increased levels of H4K16ac and γH2AX.

The exercise-dependent systemic inflammatory response^5,8,29^ that we observed immediately after a soccer game encompassed both strenuous physical activity–induced transient leukocytosis as well as increases in circulating IL-6 and TNF α, early mediators of exercise-induced inflammation^10–12^.

With regard to both the pattern and magnitude of exercise-induced changes in circulating IL-6 and TNFα concentrations, the peaks we observed in these cytokines immediately after the match, and the subsequent return to baseline values, are in accordance with other reports on the responses of both female and male soccer players^29,30^ respectively. Also, we found large positive correlations between IL-6 and TNFα as well as between IL-6 and cortisol, a steroid hormone that is the major human glucocorticoid^31^. Both IL-6 and TNFα can play additional roles during strenuous physical activity. TNFα stimulates IL-6 production and their further coordinated action encompasses, for example, glucose homeostasis and stimulation of lipolysis in adipocytes and skeletal muscle cells in response to exercise^13^. In addition to IL-6 and TNFα, the metabolic (mainly catabolic) processes that are intended to make energy available during physical effort are under the control of cortisol^9,32^. For example, cortisol, along with IL-6, stimulates lipolysis in adipocytes^31^ and the exercise-dependent release of cortisol into circulation is partially stimulated by IL-6. In fact, circulating IL-6 is an important stimulator of the hypothalamo-pituitary-adrenal (HPA) axis, leading directly or indirectly to release of cortisol from the adrenal glands^9,31,32^.

The significant increases in IL-6, TNFα, and cortisol that we observed immediately after a soccer game were accompanied by an increase in plasma lactate concentration. The post-match values that we observed were relatively low, but they were similar to those reported by Bansbo et al.^33^. Notably, changes in the lactate level during and immediately after a soccer game are highly dynamic and therefore should be considered with caution. It has been shown that the lactate level is gradually elevated in response to a number of energy-intensive activities that occur especially during the first half of a match. However, the lactate level decreases during the second half as a result of the reduced periods of high-intensity activity^1^. Moreover, marked dynamic changes in the lactate level during exercise is also a consequence of its intensified turnover during enhanced gluconeogenesis—mainly in liver—in response to physical activity. In contrast to the conventional view of lactate as a metabolic poison and a muscle fatigue factor, it also acts as a molecule of metabolic value. The lactate shuttle concept assumes that lactate is exchanged between producer and consumer cells and/or organs via blood, and hence it serves as a major energy source, the major gluconeogenic precursor, as well as an important player in cell signaling^28^.

The coordinated action of lactate and IL-6 in exercise-dependent regulation of metabolism might be reflected by the large correlation between these two variables that we observed in our study. Hojman et al.^34^ also reported a tight correlation between exercise-induced lactate production and IL‐6 release in a sample of healthy young men who performed a strenuous 2-h, interval-based cycling workout. Those authors observed these measures in electrically stimulated muscle cell cultures. Hojman et al.^34^ proposed a model that links strenuous physical activity, lactate production, and exercise-dependent release of IL-6 from muscle into circulation. This model assumes that there is a pool of IL-6 located within intramyocellular vesicle-like structures of resting skeletal muscle fibers near the sarcolemma and T-tubule regions. When strenuous exercise-induced lactate production is initiated, it activates a cascade of pH‐sensitive proteases that reside in the extracellular matrix of muscles. They, in turn, activate metalloproteases, such as matrix metalloproteinases 2 and 9 (MMP2 and MMP9, respectively), by cleaving their latent zymogenic pro‐peptides. Activation of this cascade is responsible for cleavage of the extracellular matrix and, thus, remodeling of the sarcolemma. Membrane conformational changes lead to the release of IL‐6 from intracellular stores^34^.

In the line with the data observed in this study, a coordinated action of IL-6, TNFα, and lactate—probably also involved in metabolic reprogramming immediately after a soccer game—are FTIR-based findings. We used FTIR spectroscopy combined with chemometrics for examination of biochemical profile of serum, expecting to establish infrared spectral markers associated with the soccer effort. The interesting results were related to the spectral region between 1,700–1,800 cm^−1^, assigned to the carbonyl group stretching vibrations mainly related to lipids. On the basis of principal component analysis (PCA), we found that samples collected shortly after the effort clustered distinctly compared to those gathered before and 24 h after the soccer match. The results were convincing because they explained approximately 82% of the data variability and, despite quantitative differences, there were more positive PC1 scores for every individual. These findings suggest the occurrence of temporal changes in the lipid biochemical profile shortly after soccer that lapse during the subsequent 24 h. Considering that the changes were associated with the C=O stretching vibrations, the observed temporal modifications could involve alterations in lipid biochemical profiles. To identify specific compounds that possibly contribute to the clustering pattern of the samples, we analyzed the PC1 loading factor plot, which showed that all main lipid fractions were negatively correlated with the PC1 scores. This finding means that both simple fatty acids characterized by the C=O of free carboxylic groups and the C=O of ester groups—like those in TG—were reduced in the serum shortly after the effort. Given that the area of the peak centered at 1716 cm^−1^ is larger than of that at 1733 cm^−1^^35,36^, we conclude that the temporary drop in the lipid compounds concerned more FFA than their esters. Although TG have been shown to decrease in response to a soccer match^37^, the FFA concentration is thought to progressively increase in serum during a game^3^. This phenomenon results from a higher rate of lipolysis and, consequently, a change in the choice of energy substrates (due to reduced muscle glycogen and lactate levels) that is observed in the second half of the match. It has also been suggested that during low-intensity periods of a soccer game, blood flow to adipose tissue enables the mobilization and release of FFA into the circulation^3,38^. While the previously reported declines in TG levels are in accordance with our findings, the data showing an increase in FFA immediately post-match^3^ are in opposition to our FTIR-based results. We therefore suppose that the outcome in this study might have resulted from the intensified physical activity—and thereby enhanced FFA uptake by contracting muscle—observed at the end of second half of the match. The latter was in turn forced by the situation on the pitch. The subjects played an important competitive match that day, which they actually won. Nevertheless, further studies are needed to determine exercise-dependent alterations more precise, for example, by using as material the particular serum fractions, i.e. lipids.

As mentioned above, biochemical changes that occurred immediately after the match were mainly engaged in metabolic adjustments to soccer match–related demands. In turn, 24 h later, we observed largely oxidative stress–associated changes that were accompanied by activation of the DDR pathway, as denoted by epigenetic markers, such as increased levels of H4K16ac and γH2AX.

First, we determined the ratio of the oxidized glutathione (GSSG) to its reduced state (GSH), a measure that serves as indicator of oxidative stress. The decline in GSH/GSSH ratio during the first 24 h of post-match recovery that we observed probably reflects prolonged oxidative stress and/or a decline in the scavenging action of antioxidant systems during the observation period^16^. A similar prolonged decline in GSH/GSSH ratio following a soccer match, which lasted up to 72 h, was reported by Fatouros et al.^39^. The increased levels of oxidative stress and muscle damage throughout the 72 h-recovery period was also documented by Ascensão et al.^40^. Although we noticed a significant decline in GSH/GSSG ratio following the match, we found no changes in serum concentrations of antioxidant enzymes, such as SOD1 and GPX1. Although both of these enzymes are usually located inside cells, they can also be found in the circulation^41,42^. Circulating GPX1 probably results from cell damage that leads to a leakage of intracellular proteins^41^. The occurence of circulating SOD1 is caused by hemolysis and also results from cellular secretion from peripheral tissues. In the latter case, SOD1 can be exported from cells into the circulation under normal conditions (constitutive SOD1 secretion); however, this process is significantly intensified under oxidative stress conditions (inducible SOD1 secretion). Serum SOD1 is mainly bound to circulating lipoproteins (both low- and high-density ones) and protects them from lipoperoxidation^42^. Because *SOD1* expression is regulated by many positive and negative regulatory elements whose activity is in turn regulated by a wide range of stimuli, including oxidative stress^43^, we hypothesize that soccer match-induced oxidative stress can influence the SOD1 protein level (and/or not only its activity). Nevertheless, changes in the protein level do not always reflect changes in activity and therefore are of limited value.

Among the consequences of oxidative stress is muscle cell injury, which occurs as a result of lipid peroxidation and can change the characteristics and arrangement of biological membranes or even disrupt them. Thus, lipid peroxidation can lead to leakage of intracellular proteins^19^, for example CK, which is regarded as a marker of muscle cell injury, into serum^20^. An observed in the present study raising activity of serum CK throughout 48-h-recovery period is in accordance with previous data^39,44,45^.

Interestingly, an increase in serum CK activity was accompanied by a progressive increase in plasma volume, a phenomenon called hypervolemia or hemodilution^6–8^, which was detected in this study immediately post-match and persisted for the next 24 h of recovery. The hallmarks of hypervolemia are decreases in hematocrit and hemoglobin concentrations in the absence of changes in total red blood cell volume^6–8^, all of which we observed. Although the mechanisms that underlie exercise-induced changes in plasma volume are still poorly understood, it has been suggested that it may be caused by exercise-associated muscle cell damage. The latter in turn lead to intracellular fluid to move into the extracellular space^46^ that is with line in our findings.

The negative correlations between GSH/GSSG ratio and serum CK activity and between GSH/GSSG and MDA concentration, one of the end-products of lipid peroxidation^19^, that we observed in our players suggest that muscle cell injury can be associated with oxidative stress-dependent lipid peroxidation. Although we determined the GSH/GSSG ratio in serum, one can assume that—due to the direct contact—oxidized extracellular environment (blood) had an impact on muscle cell injury.

Oxidative stress may also affect other macromolecules, such as DNA. One of the most abundant oxidative damagers of DNA is 8-OHdG. 8-OHdG is removed from DNA in healthy organisms and, unlike other species containing oxidized guanine, can pass through the cell membrane, which means it can be detected in plasma or serum^21^. An increase in serum 8-OHdG level, such as observed in this study, may also result from the release of DNA from damaged cells^47^. Other studies have also documented physical activity-induced increases in 8-OHdG concentrations^25,26^.

Overall the dynamics of the 8-OHdG and MDA changes observed in this study suggest a gradual intensification of oxidative stress-dependent damage to DNA (marked by 8-OHdG) and lipids (marked by MDA).

In addition to the aforementioned oxidation of guanine indicated by 8-OHdG, ROS-dependent damage to DNA also involves double strand breaks (DSBs). In physiological conditions DSBs are repaired because left unrepaired, they would lead to genome instability or even cell death. It is crucial, therefore, to activate molecular signaling immediately after the appearance of DSBs in order to preserve genome function and this depends heavily on chromatin modifications at the nucleosomes surrounding the DSBs, with γH2AX playing a well-established role^48^. Although the role of γH2AX in the response to DNA damage produced by a wide range of chemical and physical agents, such as high doses of ionizing radiation and oxidative stress, is well established^49^, the link between γH2AX and physical activity is still very poorly documented. While there are reports documenting an impact of physical activity on DNA damage^26^ or exercise-dependent kinetics of DNA damage repair mechanisms^25^, they focus on other, non-epigenetic components of the DDR pathway.

Our study revealed a rise in γH2AX level in PMBC in response to a soccer match, a finding which suggests that a DNA damage repair (DDR) mechanism is activated 24 h after a match. The few reports focusing on exercise-induced γH2AX foci formation include a study showing the influence of aerobic training workload on γH2AX foci parameters. Lippi et al.^50^ examined adults from the general population and trained athletes were subjected to four sequential running trials of increasing distance: 5, 10, 21 and 42 km. The values of γH2AX foci parameters increased gradually in a distance-dependent manner^50^. Another study has reported baseline levels and day-to-day variability in γH2AX foci in healthy, moderately trained subjects at rest^51^.

The formation of γH2AX foci is not the only epigenetic marker of cellular DDR, but its induction is correlated with H4K16 acetylation^22,23^. Additionally, the H4K16ac level is upregulated in response to ionizing radiation. These data suggest that this histone modification is involved in modulation of the DSB repair mechanism^52^.. Our results showing the (1) progressive increase in γH2AX level following the soccer match as well as (2) simultaneous increases in both γH2AX and H4K16ac after 24 h of recovery are consistent with these findings. Specifically, taken together these data suggest activation of an integrated, epigenetically controlled DDR pathway 24 h after the match.

Besides its role in the DDR pathway, H4K16ac is one of the most highly abundant activating modifications and serves as a molecular switch between repressive and permissive chromatin^53^. In our sample the global level of H4K16 acetylation decreased after the game, but there was a dramatic increase in its level 24 h later. This temporal pattern of H4K16ac changes suggests, in turn, that there was a decline in global transcription rate immediately after the match, although a massive increase in rate was observed at 24 h later. Zhong et al.^54^ have suggested that such a pattern might be a consequence of oxidative stress. They reported that both HCT116 and HeLa human cell lines showed an initial decrease in H4K16ac in response to acute H_2_O_2_-induced oxidative stress but recovered after 2 h of continuous H_2_O_2_ treatment^54^.

Indeed, we observed a similar dynamic pattern of H4K16ac changes, although we examined the response at organism level. In comparison with cell lines, the organism is a much more complex system and as such its homeostatic mechanisms operate at multiple levels. Nevertheless, our data seem to be consistent with the model proposed by Zhong et al.^54^. This model assumes that oxidative stress-dependent H4K16 hypoacetylation is required for suppression of DNA repair genes due to oxidative stress and may, therefore, prevent DNA damage signaling from interfering with transcription^54^. Sharma et al.^22^ also reported that reduced levels of H4K16ac correlate with repair of defective DDR and DSBs caused by ionizing radiation.

H4K16ac has also been shown to trigger release of paused Pol II. Oxidative stress results in the rapid stabilization of promoter-proximal paused Poll II throughout the human genome, and thus limits the rate of transcription^55^. Specific developmental or environmental signals are required to release Pol II from gene promoters and provide productive elongation; these in turn trigger the nucleosomal response that manifests as H4K16 acetylation^56^. We speculate that a decline in an immediate post-match level of H4K16 acetylation might be linked to oxidative stress-driven rapid stabilization of promoter-proximal paused Poll II. Consequently, a rise in H4K16ac at 24 h post-match might be associated with subsequent steps of the oxidative stress response that requires productive transcription, on a massive scale, including transcription of genes involved in the DDR pathway.

## Conclusions

One of the main findings of this study is that strenuous physical activity has an impact on changes in γH2AX and H4K16ac levels in the PMBC chromatin. We can only suggest—in light of the empirical results from relevant papers—that (1) increases in γH2AX and H4K16ac levels at 24 h post-match might result from an activation of the DDR pathway; and (2) the H4K16ac dynamics pattern could be a consequence of oxidative stress-driven delay in productive transcription. Further investigations are necessary to provide experimental mechanistic links. It would also be interesting to analyze changes in the levels of those epigenetic markers in the particular subpopulations of white blood cells, rather than the whole PMBC fraction. The latter is a limitation of this study.

Second, FTIR-based biochemical fingerprinting revealed that strenuous physical effort had the most impact on the lipid profile of serum, in particular on TG and FFA concentrations in the serum. We also showed that the ATR-FTIR technique can be applied for enhancing and integrating data obtained by traditional biochemical methods.

Finally, the wide array of correlations that we performed—although they neither provided direct mechanistic links nor explained causal relationships—accentuated some interrelations between the analyzed biochemical features, even those seemingly unrelated to each other. Some of them might, of course, be stochastic, but others might result from the same basic mechanism that involves both of the analyzed variables. Those findings, combined with the available literature data, enable us to draw some conclusions that could be explored in future studies. We believe that such a holistic approach is particularly important when considering a complex system, such as a human organism, in which the whole is greater than a simple sum of the individual parts.

## Materials and methods

### Subjects

Ten healthy, non-smoking adolescent soccer players from the Polish Central Under-19 (U-19) League, which represents the highest national level, participated in this study. During the 2017/18 season, all participants were housed in a dormitory, consumed the same diet without any nutritional supplements and followed identical training programs (12–14 h weekly). Participants and their parents were fully informed about the experimental procedures before providing written informed consent. The informed consent was obtained from all participants and their legal guardians. The study was conducted in accordance with the Declaration of Helsinki, and the protocol was approved by the Ethics Committee of the Faculty of Medicine of the University of Rzeszów, Poland (no. 3/11/2017).

### Anthropometric characteristics and body composition of the soccer players

The anthropometric characteristics of the subjects are listed in Table 2 and body composition parameters are summarized in Table 3. Anthropometric measurements were performed during a preliminary visit. Body composition parameters were determined before breakfast, one day before the match, using a Tanita MC 180 MA Body Composition Analyzer (Diagnostic Equipment Centre, Japan, Osaka).

**Table 2:** Anthropometric characteristics of the soccer players.

Parameter	Mean ± SD (N = 10)
Age [years]	17.3 (± 0.48)
Weight [kg]	70.38 (± 5.37)
Height [cm]	178.8 (± 5.85)
Body mass index [kg m^−2^]	22.09 (± 1.47)

**Table 3:** Body composition of the soccer players.

Parameter	Mean ± SD (N = 10)
Fat mass [kg]	10.50 (± 2.34)
Fat mass [%]	14.90 (± 3.03)
Free-fat mass [kg]	59.99 (± 4.92)
Free-fat mass [%]	85.10 (± 3.03)
Total body water [kg]	44.38 (± 4.12)
Total body water [%]	63.04 (± 3.65)
Muscle mass [kg]	56.94 (± 4.75)
Muscle mass [%]	80.91 (± 2.92)
Skeletal muscle mass [kg]	35.03 (± 4.21)
Skeletal muscle mass [%]	49.76 (± 4.91)
Bone mass [k]	3.00 (± 0.24)
Bone mass [%]	4.27 (± 0.19)
Predictive right leg muscle mass [kg]	10.39 (± 0.95)
Predictive right leg muscle mass [%]	79.94 (± 3.77)
Predictive left leg muscle mass [kg]	9.90 (± 1.01)
Predictive left leg muscle mass [%]	78.99 (± 4.33)

### Experimental design

On the main data collection day the subjects played a high-level competitive soccer match that ended the first round of the Polish U-19 League 2017/2018 season. The players appeared highly physically and mentally prepared. Only outfield players who played for the full 90 min. (N = 10) were included in the study. Venous blood samples were collected from participants before, immediately after and 24 h after the match, into both serum-separating and vacutainer-K_3_EDTA tubes.

### Blood sampling and processing

Serum was obtained by centrifugation (500×*g*, 20 min, RT) of blood collected in serum-separating tubes. PMBCs were isolated from blood collected in vacutainer-EDTA tubes using a histopaque-1077 (Merck, Poland), according to the manufacturer’s instructions. Aliquots of serum and PMBCs were snap-frozen in liquid nitrogen and stored at − 80 °C for later biochemical analysis and histone extraction, respectively.

Only non-hemolyzed blood samples were taken for further analysis. Blood samples were preserved from auto-oxidation and kept in the dark. To avoid inter-assay variance all assays were performed by the same technician. All samples for a particular analysis were thawed only once and assayed as a batch. Samples were assayed in duplicate.

### Analysis of exercise-induced changes in plasma volume

Percentage change in plasma volume following the soccer match was calculated from alternations in hemoglobin concentration and hematocrit values using the^57^ formula. All data were corrected for change in plasma volume as described elsewhere^57^.

### Hematological parameters measurements

Hematological parameters were determined from blood samples collected in standard vacutainer-K_3_EDTA tubes and analyzed in a clinical laboratory (Diagnostyka, Rzeszów, Poland) within 2 h of collection, using the Sysmex XE-2100D.

### Measurements of CK activity, cortisol and lactate concentration

Serum CK activity and serum cortisol and plasma lactate concentrations were determined in a clinical laboratory (Diagnostyka, Rzeszów, Poland) using Architect Ci4100. Because there are diurnal changes in cortisol concentrations all samples were collected between 7 a.m. and 10 a.m.

### Analysis of GSH/GSSG

Serum GSH/GSSG ratio was evaluated using a fluorometric kit (GSH/GSSG Ratio Detection Assay Kit, cat. no. ab138881, Abcam UK, Cambridge). Immediately after collection samples were treated with TCA to avoid auto-oxidation, then subjected to complete deproteinization (cat. no. ab204708, Abcam UK, Cambridge) according to the manufacturer’s protocol.

### Biochemical analysis

Serum levels of biochemical markers were evaluated with use of commercial ELISA kits according to the manufacturer’s instructions: IL-6 (cat. no. ab46042, Abcam UK, Cambridge), TNFα (cat. no. Ab46087, Abcam UK, Cambridge), GPX1 (cat. no. ab193767, Abcam UK, Cambridge), SOD1 (cat. no. Ab119520, Abcam UK, Cambridge), MDA (cat. no. EU2577, FineTest Ltd., China), 8-OHdG (cat no. EU2548, FineTest Ltd., China).

### Histone isolation and Western blot analysis

Core histones were extracted from PMBCs as described elsewhere^58^. Protease inhibitor cocktail (cat. no. 78429), Thermo Fisher Scientific, USA) and protease and phosphatase inhibitor cocktail (cat. no. 78446, Thermo Fisher Scientific, USA) were used during all steps of extraction and purification of the acetylated isoform of H4K16, unmodified isoform of H4, and phosphorylated isoform of H2AX (γH2AX), respectively. All protein isolation procedures were performed at 4 °C. Samples (50 μg of protein per well) were then run on 15% SDS-PAGE gel, transferred and immobilized on polyvinylidene difluoride (PVDF) membrane. Global levels of γH2AX, H4K16ac and H4 (control) were determined using commercial primary antibodies (cat. no. ab11174, ab109463, ab16483, respectively, Abcam, UK) and with secondary antibodies conjugated to HRP (Merck). The respective proteins were detected using a Clarity ECL Western Blotting Substrates (Bio-Rad) and a G:BOX imaging system (Syngene, Cambridge, UK).

All western blots were quantified by densitometry, using ImageJ program, normalized to H4 histone (a loading control as described by Bi et al.^59^), and showed as relative protein levels. All samples derived from the same experiment and blots were processed in parallel. Statistical analysis are described in the "Statistics" section.

### ATR-FTIR

ATR-FTIR was used to analyze changes in the biochemical profiles of serum following the soccer game (before the match, immediately after, and at 24 h post-match). The measurements were performed using the iZ10 module of the Nicolet iN10 MX microspectrometer (Thermo Scientific, USA) equipped with a deuterated L-alanine doped triglycine sulfate (DLaTGS) detector and KBr beam splitter and a one-bounce diamond crystal of ATR as an accessory (Smart Orbit, Thermo Scientific). The 5 μL samples were dropped on the ATR crystal and allowed to dry at room temperature to form a solid film. One hundred and twenty-eight interferograms were acquired within the wavenumber range of 4000 cm^−1^ and 400 cm^−1^ at the 4 cm^−1^ resolution and co-added. The ATR diamond crystal was cleaned carefully with ethanol or isopropanol before each measurement. The background spectra were collected before each measurement and the vapor absorption spectra collected without sample were subtracted before processing of the sample spectra. The spectra were rubber-band baseline corrected and area normalized (1,800–900 cm^−1^). Collection and preprocessing of the spectra were carried out by means of OMNIC (v. 8.1, Thermo Fischer Scientific Inc.) software. The spectral analysis and visualization were performed using ChemoSpec^60^ and hyperSpec^61^ packages in R^62^.

Chemometric analysis (PCA) was performed separately for the following spectral regions: 3,100–2,800 cm^−1^, 1,800–1,700 cm^−1^, 1,700–1,500 cm^−1^, 1,500–1,200 cm^−1^, 1,200–900 cm^−1^, and 900–600 cm^−1^ using ChemoSpec. The bands were associated to specific groups of chemical compounds or were considered as biochemical fingerprint. To resolve overlapped peaks in the loading factor curve, we performed deconvolution by employing a Gaussian function and nonlinear least-squares fitting^63^.

### Statistics

Associations between the investigated biochemical characteristics of serum were evaluated at the within-subject level. Repeated measures correlation coefficients^64^ were calculated using the rmcorr function of the rmcorr package^65^. Due to non-normality of the compared variables the *p* values, indicating the strength of the associations, were estimated on the basis of bootstrapping procedures (10,000 re-samples).

The time × effort (0 h post-effort, 24 h post-effort) effects on serum profile as well as protein levels were evaluated taking into account confidence intervals calculated for the repeated measures design and thus were referred to the within-subject level. The summary SE within function from the Rmisc package^66^ was used to remove inter-subject variability to enable estimation. The error bars and 95% confidence intervals for within-subject comparisons were adjusted according to Cousineau^67^ and Morey^68^.

Data management, statistical analysis and graphical presentation were carried out with R^62^ in R-Studio.

Effect sizes for the correlations are denoted as described elsewhere^69^: trivial < 0.10; small 0.10–0.29; moderate 0.30–0.49; large 0.50–0.69; very large 0.70–0.89; nearly perfect 0.90–0.99.

## Supplementary information

Supplementary file1
