# Supplementary material for: Changes in γH2AX and H4K16ac levels are involved in the biochemical response to a competitive soccer match in adolescent players
Source: Sci Rep. 2020 Sep 2;10:14481. doi: 10.1038/s41598-020-71436-6 (PMC7468116; doi:10.1038/s41598-020-71436-6)
Supplement: Supplementary file 1 — Supplementary file1 [file 41598_2020_71436_MOESM1_ESM.pdf]

Supplementary information

Changes in γH2AX and H4K16ac levels are involved in the biochemical response to a competitive soccer match in adolescent players

Katarzyna Kozioł[1], Jacek Żebrowski[2], Gabriela Betlej[3], Ewelina Bator[3,] Wojciech Czarny[4], Wojciech Bajorek[4], Bartłomiej Czarnota[5], Robert Czaja[4], Paweł Król[4], Aleksandra Kwiatkowska[3,*]

[1] Department of Animal Physiology and Reproduction, University of Rzeszów, Werynia 502, 36-100, Kolbuszowa, Poland.

[2] Department of Plant Physiology and Ecology, University of Rzeszów, Aleja Rejtana 16c, 35-959, Rzeszów, Poland.

[3] Laboratory of Exercise Physiology and Biochemistry, Department of Human Sciences, University of Rzeszów, Aleja Rejtana 16c, 35-959, Rzeszów, Poland.

[4] Department of Human Sciences, University of Rzeszów, Aleja Rejtana 16c, 35-959, Rzeszów, Poland.

[5] Department of Swimming Sports, University of Rzeszów, Aleja Rejtana 16c, 35-959, Rzeszów, Poland.

* Correspondence: akwiatkowska@ur.edu.pl; Tel.: +48 17 872 32 50**,** ORCID: 0000-0003-4159-977X

A

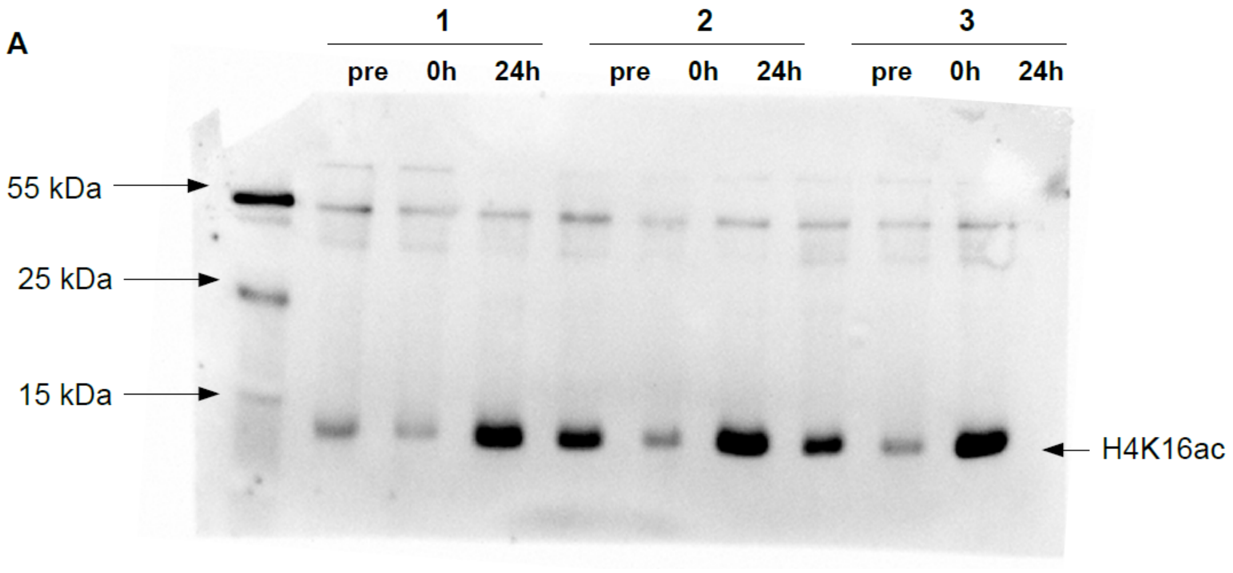
1
2
3
pre
0h
24h
pre
0h
24h
pre
0h
24h
55 kDa
25 kDa
15 kDa
H4K16ac

B

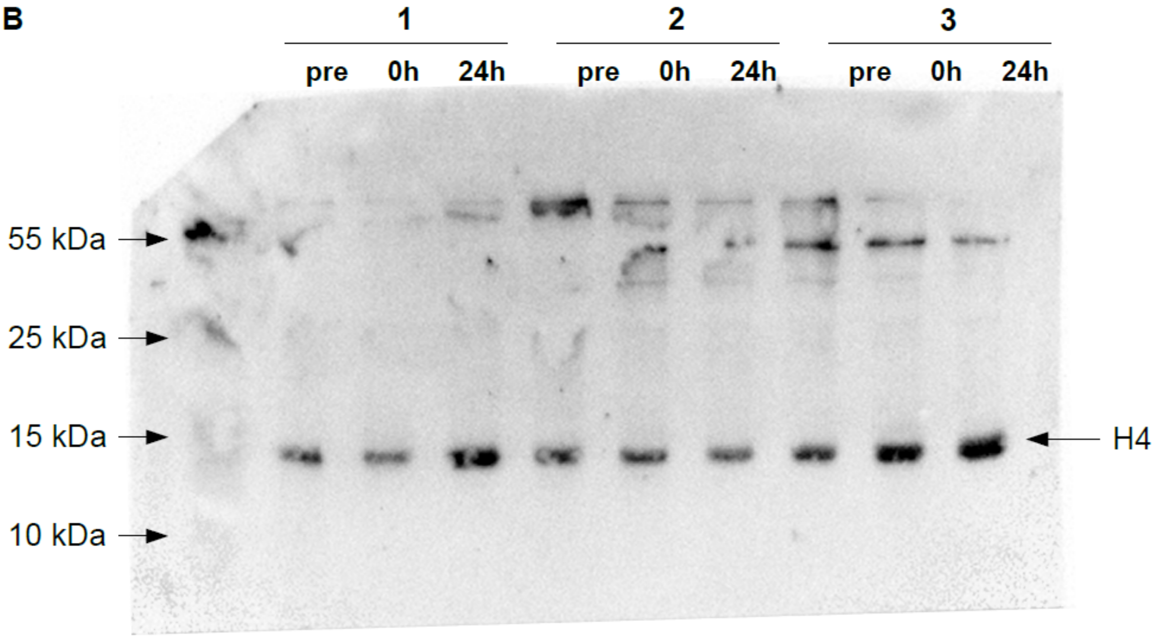
1
2
3
pre
0h
24h
pre
0h
24h
pre
0h
24h
55 kDa
25 kDa
15 kDa
10 kDa
H4

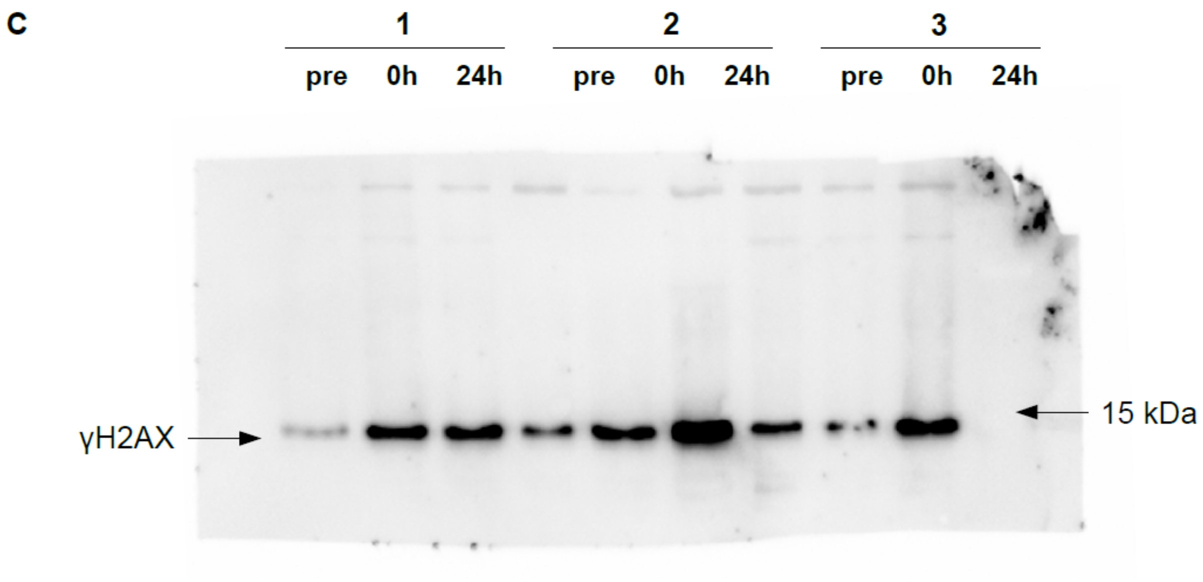

Supplementary figure S1. Full-length pictures of the western blots presented in Figure 7. **(A)** H4K16ac, **(B)** H4, **(C)** γH2AX.

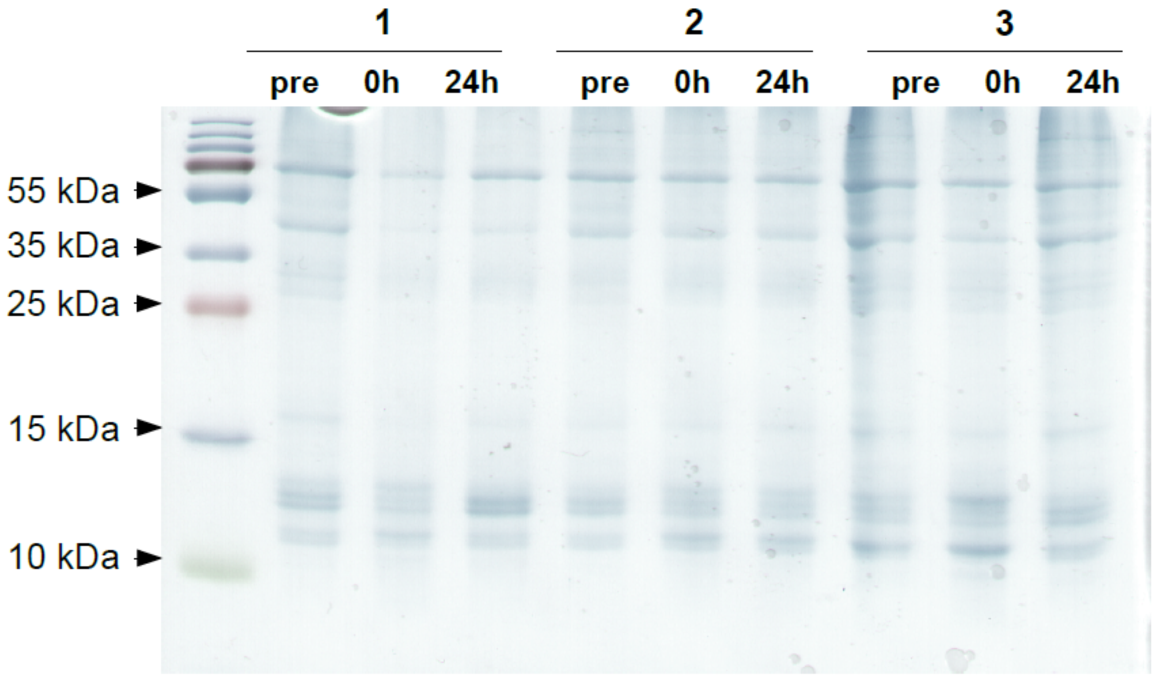

Supplementary figure S2. Full-length picture of the SDS-PAGE gel.
